# Astrocyte maturation creates a stage-specific niche that instructs hippocampal neurogenesis

**DOI:** 10.3389/fncel.2026.1966983

**Published:** 2026-10-07

**Authors:** Evangelia Masouti, Lucía Casares-Crespo, Andreas Sagner, Arne Gessner, Martin F. Fromm, Helena Mira, Ruth Beckervordersandforth

**Affiliations:** 1 Department of Neurosurgery, University Hospital Erlangen, Friedrich-Alexander-Universität Erlangen-Nürnberg, Erlangen, Germany; 2 Institute of Biochemistry, Friedrich-Alexander-Universität Erlangen-Nürnberg, Erlangen, Germany; 3 Instituto de Biomedicina de Valencia (IBV), CSIC, València, Spain; 4 Centro de Investigación Príncipe Felipe (CIPF)-Associated Unit to the Instituto de Biomedicina de Valencia (IBV), Valencia Biomedical Research Foundation, València, Spain; 5 Institute of Experimental and Clinical Pharmacology and Toxicology, Friedrich-Alexander-Universität Erlangen-Nürnberg, Erlangen, Germany

**Keywords:** astrocyte maturation, astrocyte secretome, hippocampal development, hippocampal neurogenesis, metabolomic profiling, neural stem cell fate

## Abstract

Astrocytes are a major part of the hippocampal stem cell niche and have been shown to control several steps of neurogenesis, from neural stem and precursor cell (NSPC) proliferation to the differentiation and maturation of newborn neurons. With the aim to identify new factors by which astrocytes promote neurogenesis, we isolated hippocampal astrocytes across key postnatal stages (p3, p10, and p14) and collected astrocyte conditioned medium (ACM) while keeping them for 1 week in culture. Depending on the developmental stage of the astrocytes, we uncovered a shift in the astrocyte secretome. Specifically, ACM from p3 progenitor-like astrocytes promoted a gliogenic program when applied to adult hippocampal NSPCs, while ACM derived from p10 and p14 astrocytes exhibited potent neurogenic properties. To define the molecular basis underlying the acquisition of these neurogenic properties, we performed transcriptomic profiling of astrocytes directly isolated at p3, p10 and p14 as well as astrocytes isolated at these stages that were kept in culture for 7 days. These data sets were integrated with metabolomic analysis of astrocyte-conditioned media from p3 and p14 astrocytes revealing distinct stage-specific programs enriched for secretory and metabolic pathways. Notably, we identified a combination of metabolites that accumulates as astrocytes mature and act as potent drivers of neuronal differentiation. Our findings demonstrate that astrocyte maturation establishes a metabolically and transcriptionally defined, stage-specific niche that instructs NSPC fate and drives neurogenesis.

## Introduction

Astrocytes are essential regulators of brain development, coordinating synapse formation, neurotransmitter recycling, metabolic support and radial glia-like neural stem cell (NSC) behavior (Ullian et al., 2001; Molofsky and Deneen, 2015; Chung et al., 2015; Allen and Lyons, 2018; Farhy-Tselnicker and Allen, 2018). In the developing cerebral cortex, astrogenesis follows the primary wave of neurogenesis, with embryonic radial-glia-like NSCs transitioning into progenitors that undergo extensive proliferation to fill up the neuronal and glial compartment (Malatesta et al., 2003; Noctor et al., 2002; Ge et al., 2012). Newly born cortical astrocytes undergo a gradual postnatal transition, progressively elaborating their complex morphology in direct response to the developing neuronal circuits, shifting their gene expression programs and acquiring mature physiological functions (Molofsky et al., 2012; Molofsky and Deneen, 2015; Khakh and Deneen, 2019; Bayraktar et al., 2020; Batiuk et al., 2020, Karpf et al., 2022). As in most brain regions, developmental processes such as neurogenesis and astrogenesis are completed, and only persist in the so-called adult neurogenic niche, the dentate gyrus (DG) of the hippocampal formation and the subependymal zone of the lateral ventricle (Götz and Huttner, 2005; Ming and Song, 2011).

Starting slightly later than the neocortex, the DG is formed from late embryonic stages onwards, proceeding into the first postnatal weeks. Neurogenesis is the initial process, whereas astrogenesis starts after birth, and the two processes continue to run simultaneously lifelong (Bond et al., 2020; Berg et al., 2019; Schneider et al., 2022). At postnatal day 3 (p3), the DG is dominated by extensive proliferation of radial glia-like NSCs, which have generated very little neuronal and astrocytic progeny so far. By p7, the NSC pool has decreased, while neuronally determined NSPCs expand and the first few astrocytes appear in the superficial DG layers. Between p10 and p14, NSCs continue to decline as the neuronal compartment expands steadily, and astrocytes increase in number, progressively occupying the deeper DG layers. Hence, the neuronal compartment expands significantly during the first postnatal week, whereas astrogenesis becomes substantial from the second postnatal week onwards. Notably, developing astrocytes follow the same outside-in pattern described for granule neurons, meaning that astrocytes occupy the DG only after neurons are already present (Mathews et al., 2010; Schneider et al., 2022; Chalmers et al., 2024). As a consequence, developing astrocytes are born into an environment which is shaped by already existing neurons. Like in the neocortex, the morphological expansion of astrocytes as well as the establishment of their individual domains occurs in parallel to extensive synaptogenesis and circuit formation in the hippocampus (Ullian et al., 2001).

Astrocytes influence neurogenesis and neuronal differentiation and maturation through a wide range of contact-mediated and secreted signals (Barkho et al., 2006; Schneider et al., 2019). Classical astrocyte-derived molecules, including thrombospondins, HEVIN, SPARC, glypicans, and cholesterol-associated lipoproteins promote synapse formation and maturation (Christopherson et al., 2005; Eroglu et al., 2009; Kucukdereli et al., 2011; Allen et al., 2012). Astrocytes also release cytokines and growth factors that modulate radial glial-like NSC proliferation and differentiation (Song et al., 2002). In addition to these protein-based cues, metabolites such as lactate, ketone bodies, and lipids have emerged as important mediators of neuron–glia communication, influencing radial glia-like NSC fate decisions as well as neuronal differentiation and synapse formation (Magistretti and Allaman, 2015; Knobloch et al., 2017), however, the contribution of metabolites in neurogenesis is not fully understood yet. Importantly, comparisons across CNS regions have shown that astrocytes from non-neurogenic regions, such as adult spinal cord, lack many of these pro-neurogenic cues, indicating that astrocyte identity and secretome are tightly linked to developmental stage and local environment (Song et al., 2002; Lie et al., 2005; Lattke et al., 2021). During early postnatal development, however, the hippocampal formation as a whole is still undergoing neurogenesis and astrogenesis, such that hippocampal astrocytes mature within an environment in which neuronal circuits are simultaneously forming (Bond et al., 2020). Defining how developing astrocytes shape their metabolic microenvironment is therefore essential to understand hippocampal niche maturation.

In this study, we set out to define how hippocampal astrocytes mature molecularly across postnatal development, and how their developmental stage shapes the signals they release to influence radial glia-like NSC behavior. To do so, we established a method to isolate astrocytes from key developmental time points (p3, p10 and p14), which correspond to progenitor-like, early-mature and mature astrocytes *in vivo* (Schneider et al., 2022). Combining functional assays with transcriptomic and metabolomic profiling, we characterize how astrocytes and their secreted factors change across postnatal development, and how stage-specific secreted metabolites influence neuronal differentiation in an *in vitro* model of hippocampal neural stem and precursor cells (NSPCs). This integrated approach allowed us to identify novel astrocyte-derived metabolites that modulate neuronal differentiation, and to provide a valuable resource of astrocytic factors shaping the process of neurogenesis.

## Materials and methods

### Animals

All animal procedures were conducted in accordance with Directive 2010/63/EU and applicable to German animal-welfare regulations. Experimental protocols were approved by the Government of Middle Franconia and complied with institutional and national guidelines for the care and use of laboratory animals. All efforts were made to minimize the number of animals used and reduce their suffering. C57BL/6 J mice of either sex were used for all experiments. p3, p10 and p14 pups were used for astrocyte isolation, and adult (8–12 weeks old) mice were used for NSPC cultures where indicated. Animals were housed under standard 12 h light/dark conditions with ad libitum access to food and water.

### Primary NSPC culture

Neural stem/progenitor cells (NSPCs) were isolated from the dentate gyrus (DG) of adult mice (8–12 weeks old). DG were microdissected in ice-cold HBSS (Life Technologies) and transferred to 15 mL Falcon tubes on ice. Tissue was enzymatically dissociated in the dissociation solution consisting of 1x HBSS supplemented with 30 mM D-glucose (Sigma-Aldrich), 15 mM HEPES (Life Technologies) (pH 7.5), trypsin (Sigma-Aldrich, T4665) and hyaluronidase (Sigma-Aldrich, H3884). Samples were incubated in 5 mL of this dissociation solution for 15 min at 37 °C, gently triturated ten times with a glass pipette and returned to 37 °C for additional 15 min, ensuring that the total incubation time did not exceed 30 min. The reaction was stopped by adding an equal volume of EBSS (Life Technologies) containing 4% BSA (Sigma-Aldrich, A4503) and 20 mM HEPES (pH 7.5). The suspension was passed through a 70-μm strainer (Miltenyi Biotec, 130-098-462) and centrifuged at 300g for 10 min. The supernatant was removed and the pellet was resuspended in 10 mL of ice-cold solution 0.5x HBSS containing 0.9 M sucrose (Sigma-Aldrich). Cells were centrifuged at 400g for 15 min, the supernatant was removed and the final pellet was resuspended in neurosphere proliferation medium containing DMEM/F12 Glutamax (Life Technologies) supplemented with 2% Neurobrew-21 (Miltenyi Biotec), 1% penicillin/streptomycin (Life Technologies), 8 mM HEPES and 1 μg/mL of the growth factors EGF and bFGF (Peprotech, AF-100-15 and AF-100-10B). Cells were plated at a density of 10.000 cells/mL in 25 cm^2^ or 75 cm^2^ tissue culture flasks (Greiner Bio-One). Cultures were maintained as neurospheres at 37 °C and 5% CO2. NSPCs were passaged approximately once per week. Neurospheres were collected and centrifuged at 500 rpm for 5 min. Pellets were resuspended in 1 mL Accutase (Sigma Aldrich, A6964) and incubated at 37 °C for 5–10 min. Cells were dissociated to a single-cell suspension by gentle trituration, diluted in 5 mL PBS (Life Technologies) and centrifuged at 1500 rpm for 5 min. The pellet was resuspended in neurosphere proliferation medium and replated at specific cell densities described above.

### MACS-based astrocyte isolation

Astrocytes were isolated from postnatal mice (p3, p10 and p14) using the Neural Dissociation Kit (P) (Miltenyi Biotec, 130–092-628) in combination with the Anti-ACSA-2 Microbead Kit (Miltenyi Biotec, 130-097-678). For each age group, hippocampi from approximately 6–8 mice were pooled, yielding 6–10 wells of primary astrocyte cultures in 24-well plates. Hippocampi were dissected in ice-cold HBSS without Ca^2+^/Mg^2+^ (Sigma Aldrich, 5501C) under a stereomicroscope. Tissue was weighed in 1 mL HBSS without Ca^2+^/Mg^2+^ and transferred to a gentleMACS C-Tube (Miltenyi Biotec, 130-093-237) containing 1950 μL enzyme mix 1 and 30 μL enzyme mix 2 (Neural Dissociation Kit (P)). Tubes were sealed, inverted and processed on a gentleMACS Octo Dissociator with Heaters using the program 37 °C_NTDK_1. Following dissociation, tissue was further triturated using a flame-polished glass pipette. The suspension was diluted with 10 mL HBSS with Ca^2+^/Mg^2+^ (Sigma Aldrich, 550037C) and passed through a 70-μm strainer (Miltenyi Biotec, 130-098-462). Cells were centrifuged at 300g for 10 min at 4 °C and the supernatant was removed. For the magnetic labeling, the pellet was resuspended in 80 μL AstroMACS Separation buffer (130-117-336) per 10^7^ total cells. FcR blocking reagent (10 μL per 10^7^ cells) was added and incubated for 10 min at 2–8 °C, followed by 10 μL Anti-ACSA-2 Microbeads per 10^7^ cells and an additional 15 min incubation. Cells were washed with 1–2 mL buffer per 10^7^ cells and centrifuged at 300g for 10 min. The pellet was resuspended in 500 μL buffer and applied to an MS column (Miltenyi Biotec, 130-042-201) placed in a magnetic separator. The column was washed three times with 500 μL buffer, and unlabeled cells were collected and discarded. The column was then removed from the magnet, placed on a clean tube, and ACSA-2+ astrocytes were eluted with 1 mL buffer. Cells were counted and pelleted at 300g for 10 min. The pellet was either resuspended in RNAlater solution (Life Technologies) and stored at −80 °C for downstream analysis (direct samples) or diluted in culture medium for immediate use (cultured samples) as described below.

### Primary astrocyte culture

Glass coverslips in 24-well plates were coated with 0.01% poly-L-lysine (Sigma Aldrich, P9155) overnight at 37 °C, washed three times with double-distilled water, and air-dried under sterile conditions immediately before use. ACSA-2^+^ astrocytes were resuspended in pre-warmed AstroMACS Medium (Miltenyi Biotec, 130-117-031, prepared by supplementing MACS Neuro Medium with 0.2% AstroMACS Supplement, 2% MACS Neurobrew-21 and 0.25% L-Glutamine (0.5 mM)) and adjusted to 50.000–80.000 cells in 50 μL. The cell suspension was placed as a central drop into each coverslip and incubated for 30 min at 37 °C to allow cell attachment. Subsequently, 450 μL of medium was gently added to each well. Cultures were maintained at 37 °C and 5% CO_2_ and 50% of the medium was replaced every other day. Astrocyte conditioned medium (ACM) was collected, centrifuged and filtered to remove debris, aliquoted and stored at −80 °C for further experiments. After 7 days in culture astrocytes were either fixed in 2% paraformaldehyde (PFA) for immunostaining or harvested for RNA isolation and stored in RNAlater solution at −80 °C.

### RNA isolation

Total RNA was isolated using the RNeasy Plus Mini Kit (Qiagen, 74,134) according to the manufacturer’s instructions. Cells preserved in RNAlater were thawed on ice, pelleted at 5.000 g for 5 min at 4 °C, and the supernatant was removed prior to lysis. Cell pellets were lysed in RLT Buffer, passed through the gDNA Eliminator column to remove genomic DNA, mixed with 70% ethanol, and processed on RNeasy Mini spin columns. After the recommended washing steps, RNA was eluted in 12–25 μL RNase-free water. RNA concentration and purity were determined using a Nanodrop spectophotometer and samples were stored at −80 °C until further use.

### Primary adult NSPC differentiation with astrocyte conditioned medium (ACM)

Adult primary NSPCs were split as usual and seeded into PDL/laminin-coated glass coverslips in 24-well plates at a density of 100.000 cells per well. Cells were initially maintained in NSPC proliferation medium containing the growth factors EGF and bFGF. After 24 h, the medium was replaced with differentiation medium lacking growth factors. Experimental conditions included treatment with ACM collected from p3, p10, or p14 astrocyte cultures as described before, while NSPC differentiation medium served as the positive control and fresh non-conditioned astrocyte medium as the negative control. At day *in vitro* 5, cells were fixed with 2% PFA for 10 min at room temperature, washed with PBS and stored at 4 °C until further processing.

### Metabolite treatment of primary adult NSCs during differentiation

Adult primary NSPCs were plated into PDL/laminin-coated glass coverslips and transitioned to differentiation medium as described above. Metabolites were added directly to 500 μL differentiation medium per well to achieve the desired final concentrations. Arachidonic acid (AA) and eicosapentaenoic acid (EPA) were applied at 1–10 μΜ (AA) and 5–50 μΜ (EPA), including combination treatments (5/5 μΜ, 10/10 μΜ and 5/10 μΜ (EPA/AA)). Cholesterol sulfate (CS) was applied at 10–50 μΜ and L-DOPA at 10–100 μΜ. All metabolites except for L-DOPA were diluted in ethanol, while L-DOPA was diluted in acidified water. Corresponding vehicle controls (ethanol and acidified water) were incubated at matched volumes. At day *in vitro* 5, cells were fixed in 2% PFA and stored as described above.

### Immunocytochemistry

PFA fixed cells were washed with PBS and incubated with primary antibodies diluted 1:1.000 in blocking solution (PBS containing 3% donkey serum and 0.5% Triton-X100) overnight at 4 °C. The following primary antibodies were used: mouse anti-MAP2(a + b) (Sigma Aldrich, AB477171), rabbit anti-GFAP (Agilent, Z033429-2) and guinea pig anti-DCX (Merck, AB2253). After washing with PBS, cells were incubated for 2 h at room temperature with species-appropriate secondary antibodies (Alexa Fluor 488, Cy3 or Cy5 conjugates) diluted 1:1.000 in blocking solution. Coverslips were then incubated with DAPI (Life Technologies, 1:10.000 in PBS) for 10 min, washed twice with PBS and mounted using Aqua-PolyMount (Polysciences).

### RNA-seq library preparation and data processing

Total RNA integrity was assessed using the Bioanalyzer 2.100 (Agilent Technologies). Library preparation and sequencing were performed by Novogene. Briefly, mRNA was purified using poly-T oligo-attached magnetic beads, fragmented and reverse-transcribed using random hexamer primers. Strand-specific or non-strand-specific libraries were generated following Novogene standard protocols, and library quality was confirmed by Qubit, qPCR and Bioanalyzer analysis. Libraries were pooled according to effective concentration and sequenced on an Illumina platform using paired-end Sequencing-by-Synthesis chemistry. Raw reads were processed using fastp for adapter trimming and quality filtering. Clean reads were aligned to the *Mus musculus* reference genome (GRCm39) using HISAT2 (v2.0.5). Gene-level counts were generated using featureCounts (v1.5.0-p3). Raw gene-level counts were used for differential expression analysis. FPKM values were used to define expressed genes (FPKM>1) and to generate the two Venn diagrams, whereas raw count matrices were used for DESeq2 differential-expression and downstream GO enrichment analyses.

### RNA-seq bioinformatic analysis

Gene-level raw count matrices were imported into R for downstream analysis. Directly isolated astrocytes and cultured astrocytes were analyzed separately for condition-specific analyses. Each group included p3, p10 and p14 developmental stages. Genes with zero total counts across all the samples were removed. Raw count matrices were analyzed using DESeq2 for normalization, dispersion estimation and differential expression. For each developmental stage, comparisons were performed against each of the other two stages. Stage-specific genes were retained when they met the significance criteria in both comparisons. *p*-values were adjusted using the Benjamini-Hochberg procedure. Genes with adjusted *p*-value<0.05 and an absolute log_2_FoldChange > 0.5 were considered differentially expressed. Both upregulated and downregulated genes were included. Functional enrichment analysis was performed using clusterProfiler, including Gene Ontology (GO). All visualizations, including PCA, volcano plots, heatmaps, bar plots and dot plots were generated using ggplot2 and appropriate visualization packages.

### Metabolomics profiling

Untargeted metabolomics was performed by the Core Unit Metabolomics of the Medical Faculty of the Friedrich-Alexander-Universität Erlangen-Nürnberg (FAU). Astrocyte conditioned medium (ACM) samples were collected after 7 days *in vitro* along with AstroMACS Medium incubated for the same duration without cells. Samples were prepared with the addition of recovery and internal standards as previously described in detail (Gessner et al., 2023). In brief, samples were analyzed using high resolution mass spectrometry on a Q Exactive™ Focus system (Thermo Fisher Scientific, Dreieich, Germany) coupled to liquid chromatography (Dionex Ultimate™ 3000, Thermo Fisher Scientific, Dreieich, Germany). Raw data, QC samples, and blanks were processed using Compound Discoverer (CD) (Thermo Fisher Scientific, Dreieich, Germany). Metabolite features were preliminary annotated using Compound Discoverer and cross-checked with an in-house library of reference standards. To focus on metabolites actively secreted into ACM, features were filtered using log_2_FoldChange ≥ 2 relative to control medium. Statistical significance was defined as adjusted *p* < 0.05.

### Metabolomics bioinformatic analysis

All downstream metabolomics analyses were performed in R. Metabolite names were aligned across detection modes and annotation inconsistencies were resolved using MetaboAnalyst and custom R scripts.

### Image analysis and quantification

All images were acquired using a Zeiss inverted Axio Observer 7 Apotome.2 equipped with an Axiocam 503, a Colibri 7 LED light source and 20x objective. For each condition, 5–10 randomly selected fields per coverslip were imaged under identical exposure settings to ensure comparability across samples. Images were processed and analyzed in Fiji Image J. Marker-positive cells were quantified manually and expressed relatively to the total number of DAPI-positive nuclei, with 100% corresponding to all DAPI-labeled cells and each marker reported as a percentage of this total. All imaging and quantifications were performed in a blinded manner.

### Statistical analyses

All statistical analyses were performed in R. Quantification of *in vitro* assays was analyzed using Student’s *t*-tests. RNA-seq differential expression was analyzed using DESeq2. For metabolomics, statistical comparisons were performed in Compound Discoverer, and downstream analyses were carried out in R. Statistical significance was defined as adjusted *p* < 0.05 unless otherwise specified.

## Results

### Astrocytes from early to late postnatal stages exhibit a differential capacity to promote neurogenesis

In the postnatal hippocampus, neurogenesis and astrogenesis are parallel processes which conjoinedly assemble hippocampal neural circuitries (Bond et al., 2020; Schneider et al., 2022). There is evidence that astrocytes actively shape distinct developmental steps of the neurogenic lineage by the release of specific factors (Schneider et al., 2019). Here, we hypothesized that astrocytes from different postnatal days may offer a cocktail of secreted factors which is specifically tailored to the stage of hippocampal development. To test this, we prospectively isolated ACSA2^+^ hippocampal astrocytes by magnetic activated cell sorting (MACS) from p3, p10 and p14 mice, representing progenitor-like, early-mature and mature astrocytes (Schneider et al., 2022). MACS-isolated astrocytes were cultured for 7 days in serum-free medium (AstroMACS Medium, Miltenyi). Exposure to serum can substantially alter the astrocyte’s morphology as well as transcriptional states (Foo et al., 2011), while serum-free conditions preserved the characteristic ramified astrocyte morphology and prevented serum-induced reactive changes, thus maintaining an *in vivo*-like state (Caldwell et al., 2022) (Supplementary Figure 1A). To assess the stage-specific secretomes, astrocyte conditioned medium (ACM) was collected throughout the culture period by exchanging half of the medium every other day. To test a stage-specific influence on neurogenesis, we isolated hippocampal NSPCs from adult wildtype mice, and kept them in neurosphere medium containing growth factors (NSPC proliferative medium) for three passages, before we forced them to undergo differentiation by withdrawal of growth factors (NSPC differentiation medium). This medium is optimized for neuronal differentiation, but its content is not known. Instead of applying NSPC differentiation medium, we cultured the NSPCs in the ACM from p3, p10 and p14 astrocytes for 5 days. After that, the cells were fixed and immunofluorescence stainings were performed against markers for mature neurons (MAP2), immature neurons (DCX) and astrocytes (GFAP), to determine the rate of neuronal versus astrocytic differentiation (Figure 1A). As controls, NSPCs were cultured either in non-conditioned AstroMACS Medium (8.98% of MAP2^+^ neurons, 73.94% GFAP^+^ astrocytes; Figures 1B,G–I) or in NSPC differentiation medium (57.98% of MAP2^+^ neurons, 26.80% GFAP^+^ astrocytes; Figures 1C,G–I). ACM from p3 astrocytes resembled the negative control condition, giving rise to mostly GFAP^+^ cells (74.20%) and little neurons (9.08%; Figures 1D,G). In contrast, ACM from p10 and p14 astrocytes substantially increased neuronal differentiation and maturation. P10 ACM yielded 51.79% MAP2^+^ neurons and 35.92% GFAP^+^ astrocytes; Figures 1E,H) and ACM from p14 astrocytes produced the highest MAP2^+^ fraction (62.85%) and even less GFAP^+^ astrocytes (23.72% Figures 1F,I). Notably, the proportion of DCX-positive immature neurons remained unchanged across all conditions. These findings suggest that astrocytes from the second postnatal week secrete factors that promote neuronal differentiation of adult hippocampal NSPCs, while astrocytes from the first postnatal week foster a gliogenic program, indicating a developmental shift in their functional properties.

**Figure 1:**
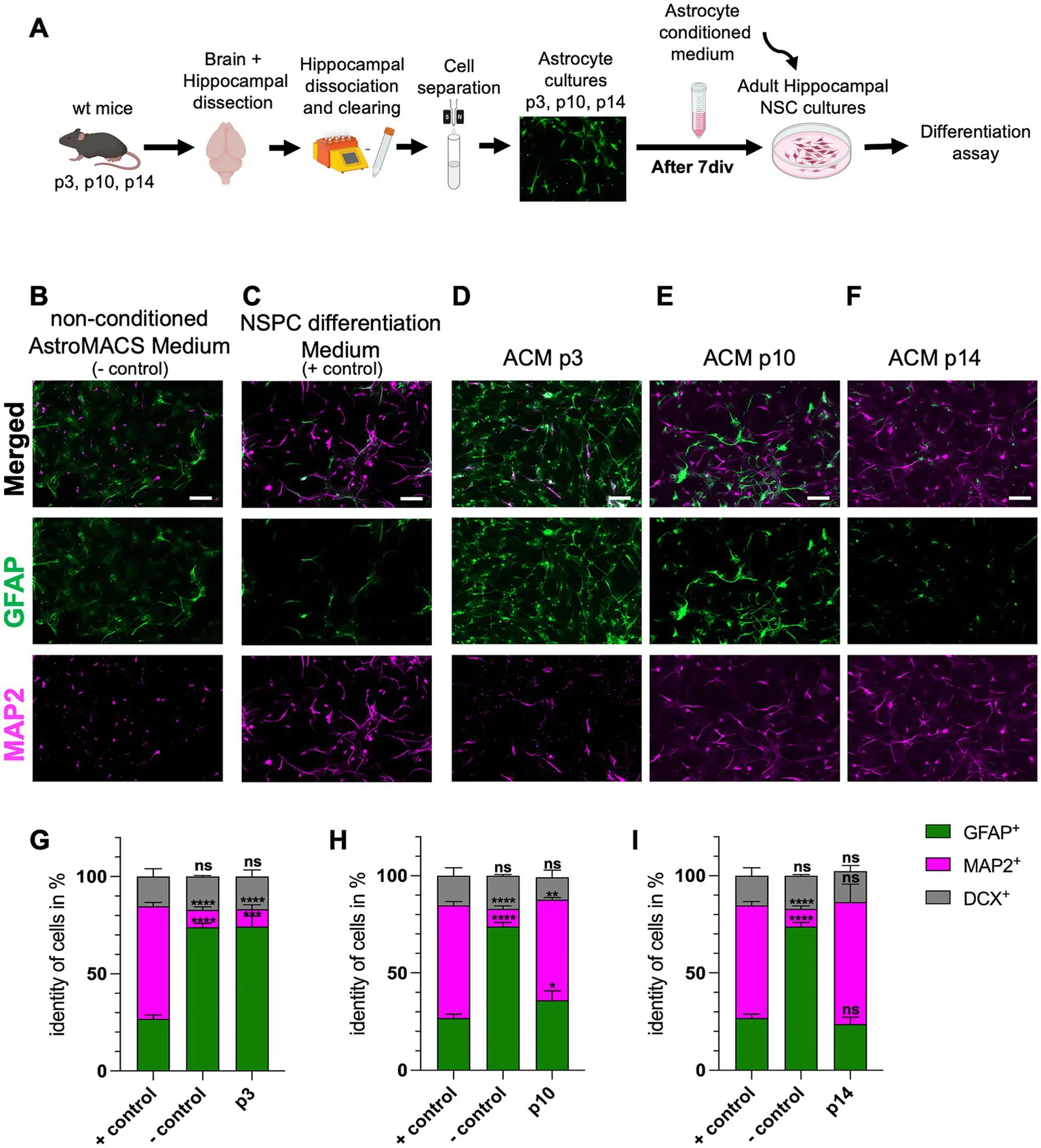
Hippocampal astrocytes isolated at distinct developmental stages differentially instruct adult NSPC differentiation. **(A)** Schematic of the experimental workflow. Hippocampal astrocytes were isolated at p3, p10 and p14, enriched using ACSA2 magnetic beads, and cultured for 7 days in serum-free AstroMACS Medium. Astrocyte conditioned medium (ACM) was collected and applied to cultured adult hippocampal NSPCs undergoing differentiation for 5 days. **(B–F)** Representative immunostainings and quantification of differentiated NSPCs under the indicated conditions. Cells were stained for MAP2, GFAP, DCX and DAPI. **(B)** Non-conditioned AstroMACS medium. **(C)** NSPC differentiation medium **(D)** p3 ACM. **(E)** p10 ACM. **(F)** p14 ACM. **(G–I)** Quantification of MAP2-positive, GFAP-positive and DCX-positive cells for each condition. Scale bars = 50 μm. Masouti, E. (2026) https://BioRender.com/9rx83j7

### Bulk RNA-seq reveals shared and stage-specific transcriptional programs in directly isolated hippocampal astrocytes across early postnatal development

To characterize the transcriptional programs underlying postnatal astrocyte identity, we performed bulk RNA sequencing on directly isolated hippocampal astrocytes at p3, p10 and p14 (referred to as p3direct, p10direct and p14direct, respectively). Astrocytes were prospectively isolated using ACSA2 magnetic beads, and freshly isolated cell pellets were processed immediately for RNA isolation and sequencing (Figure 2A) to capture their stage-specific transcriptional state without culture-induced alterations.

**Figure 2:**
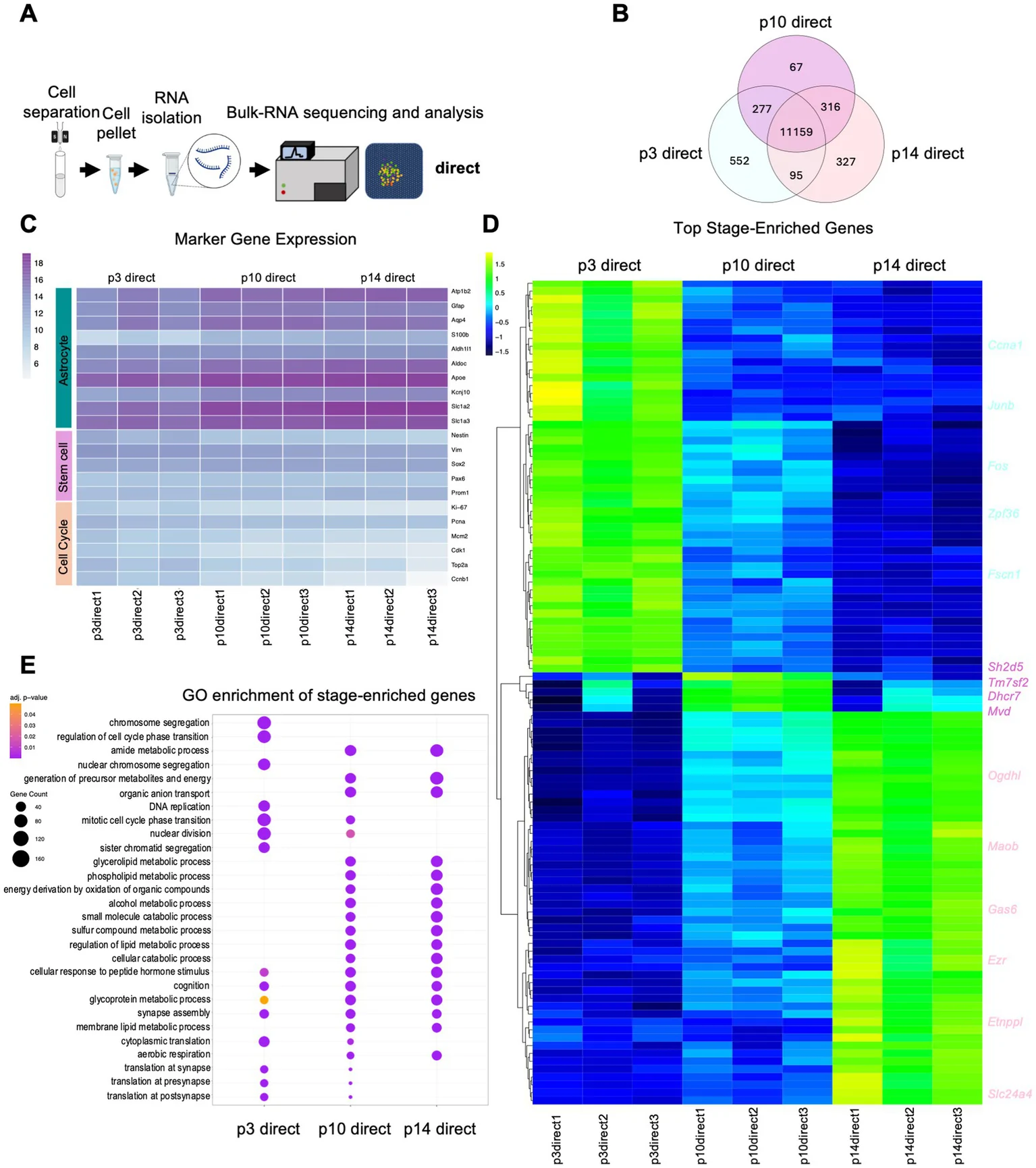
Shared and stage-enriched transcriptional programs in directly isolated hippocampal astrocytes across postnatal development. **(A)** Schematic of the experimental workflow. Hippocampal astrocytes were enriched using ACSA2 magnetic beads, and freshly collected cell pellets were processed for RNA extraction and bulk RNA sequencing. **(B)** Venn diagram showing shared and stage-specific expressed genes across p3, p10 and p14 astrocytes. **(C)** Heatmap depicting log_2_ expression of astrocyte, stem cell and cell cycle markers across p3, p10 and p14 directly isolated astrocytes. **(D)** Heatmap presenting the top stage-enriched genes for each developmental stage. **(E)** Bubble plot showing Gene Ontology (GO) enrichment for stage-enriched gene sets at p3, p10 and p14. Masouti, E. (2026) https://BioRender.com/9rx83j7

Astrocytes shared a large core transcriptome consisting of 11.159 genes across p3, p10 and p14 (Figure 2B), and Gene Ontology (GO enrichment) analysis revealed persistent biosynthetic and astrocytic-lineage programs, including RNA metabolism, protein localization, mitochondrial organization, phospholipid metabolism, alongside neural-related categories such as synapse organization, regulation of neurogenesis and astrogenesis (Supplementary Table 1). Together, these shared processes define a stable molecular backbone that remains conserved throughout hippocampal postnatal development.

To assess the identity of the directly isolated astrocyte populations, we examined the expression of core astrocyte, stem cell and cell cycle markers (Figure 2C). Log_2_-normalized expression revealed high levels of core astrocyte markers, including *Atp1b2, Gfap, Aqp4, Aldh1l1, Aldoc, Apoe, Slc1a2* and *Slc1a3*, across all samples, confirming robust astrocyte enrichment across all stages. *S100β* displayed lower expression at p3 and increased at later stages. Stem cell-associated genes such as *Vimentin (Vim), Sox2, Pax6* and *Prom1* were consistently low. *Nestin*, however, was elevated at p3, reflecting the expected presence of stem cells and glial progenitor cells at this early developmental stage. Cell cycle markers including *Ki-67, Mcm2, Cdk1, Top2a* and *Ccnb1* were also higher at p3, reflecting increased proliferative activity. Together, these expression profiles resembled the expected identity and developmental state of the directly isolated astrocytes.

To distill out the molecular fingerprints of p3, p10 and p14 directly isolated astrocytes, we next assessed stage-enriched differential expressed genes (DEGs). Here, we observed clear developmental distinctions (Figure 2D): while p3 astrocytes were defined by early-response and cell cycle-associated genes, including *Junb, Fos, Egr2, Zpf36, Ccna1* and *Fscn1*, p14 astrocytes exhibited a more mature transcriptional profile, expressing genes such as *Ogdhl, Maob, Etnppl, Ezr, Gas6* and *Slc24a4*. Interestingly, p10 astrocytes showed comparatively few stage-enriched DEGs and appear to represent an intermediate transcriptional state. Several genes associated with the p3 and p14 signatures were expressed at lower levels in p10 astrocytes. To place these gene-level differences into a broader functional framework, we next compared GO enrichment across stages (Figure 2E). Consistent with their proliferative identity, p3 astrocytes were significantly enriched for mitotic and DNA replication pathways. In contrast, p10 and p14 astrocytes shared many biological processes including lipid metabolic processes, synapse-related pathways and oxidative metabolism, highlighting a substantial functional overlap between these two stages. However, p10 astrocytes showed greater enrichment for lipid and fatty acid metabolic programs and synaptogenic pathways, whereas p14 astrocytes exhibited higher enrichment for oxidative phosphorylation, aerobic respiration, and small molecule catabolism.

Together, these data reveal a coordinated transcriptional progression in which astrocytes transition from a proliferative state at p3, marked by genes such as *Cenpf, Mcm7* and *Cdh1*, through a metabolically shifting and synaptogenic phase at p10, characterized by genes such as *Elvovl5, Scd1* and *Sh2d5* to a mature mitochondrial and neuro-protective state by p14, defined by genes including *Cox6a2, Idh3a* and *Etnppl*.

### Bulk RNA-seq reveals shared and stage-specific transcriptional programs of cultured hippocampal astrocytes

To determine how the molecular programs of astrocytes from different developmental stages adapt to cell culture conditions and to identify stage-specific factors that may influence the process of neurogenesis, we performed bulk RNA-seq on hippocampal astrocytes from p3, p10 and p14 mice after culturing them for 7 days (referred to as p3cultured, p10cultured and p14cultured, respectively; Figure 3A).

**Figure 3:**
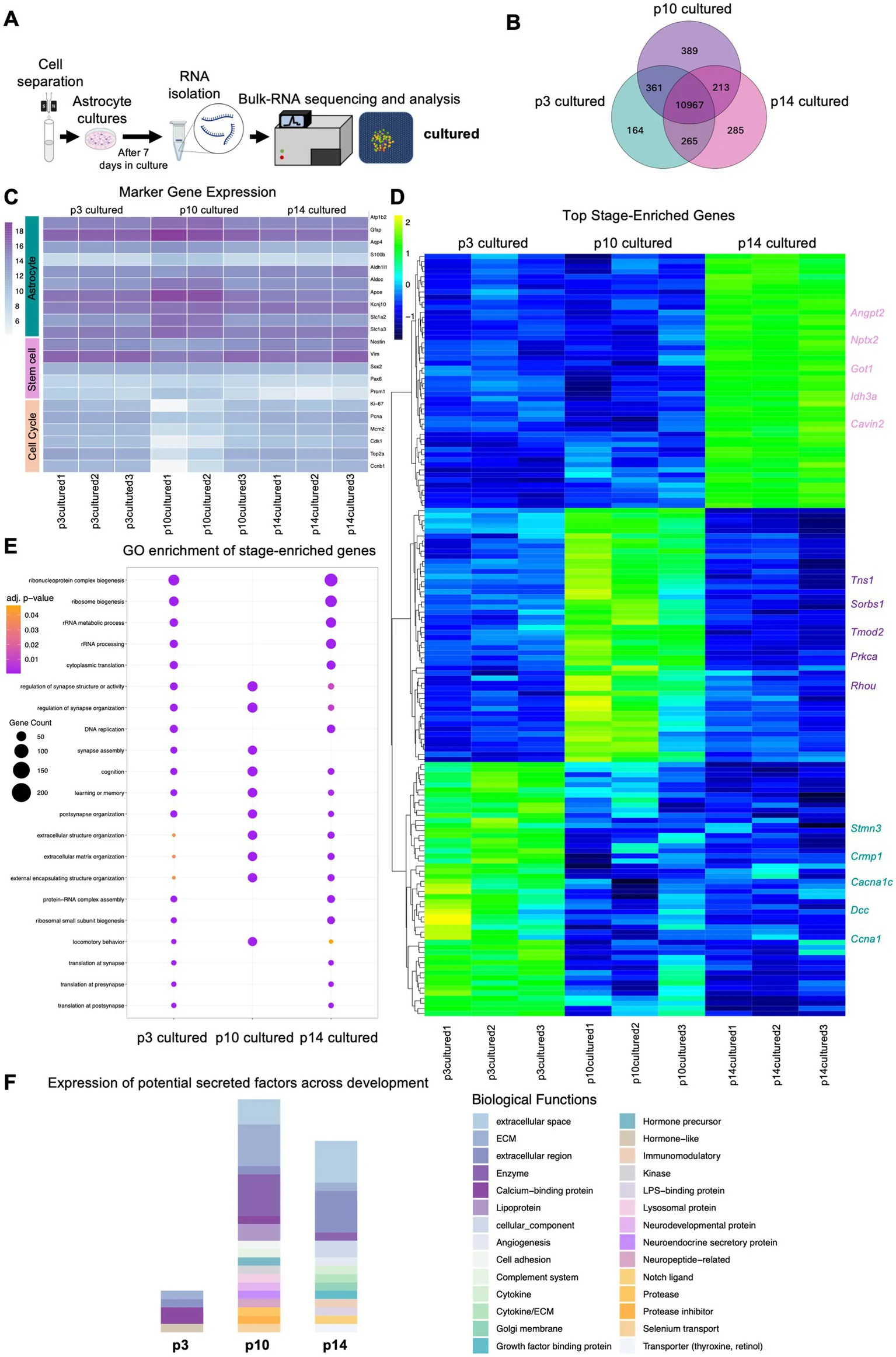
Transcriptional programs of cultured hippocampal astrocytes across postnatal development. **(A)** Schematic of the experimental workflow. Hippocampal astrocytes were enriched using ACSA2 magnetic beads and maintained for 7 days in culture prior to bulk RNA sequencing. **(B)** Venn diagram showing shared and stage-specific expressed genes across p3, p10 and p14 astrocytes cultured astrocytes. **(C)** Heatmap depicting log_2_ expression of astrocyte, stem cell and cell cycle markers across p3, p10 and p14 cultured astrocytes. **(D)** Heatmap showing the top stage-enriched genes. **(E)** Bubble plot illustrating GO enrichment for stage-enriched gene sets at p3cultured, p10cultured and p14cultured. **(F)** Stacked bar plot depicting stage-specific functional categories of potential secreted factors identified from the cultured astrocyte transcriptomes. Masouti, E. (2026) https://BioRender.com/9rx83j7

We first examined to which extent cultured astrocytes share transcriptional programs across developmental origins. All astrocytes retained a large core transcriptome, consisting of 10.967 genes across all three stages (Figure 3B). GO enrichment of this shared gene set revealed strong enrichment for core cellular processes, including ribonucleoprotein complex biogenesis, RNA processing, rRNA metabolic process and regulation of protein stability (Supplementary Table 2). Additional enriched categories reflected metabolic and organelle-associated pathways, such as mitochondrion organization, mitochondrial gene expression and regulation of autophagy. Notably, unlike the directly isolated astrocytes, the shared cultured transcriptome also included cell cycle and chromosome dynamics pathways, indicating that culture conditions induce proliferative and structural remodeling signatures that are not present in the directly isolated samples. Despite these culture-specific features, the shared gene set retained glial identity programs including gliogenesis. Together, these findings show that, although cultured astrocytes retain a large conserved molecular backbone, they also acquire culture specific molecular signatures that are absent from all directly isolated samples.

To assess the purity and developmental state of the cultured astrocyte populations, we evaluated the expression of core astrocyte, stem cell and cell cycle markers (Figure 3C). Core astrocyte markers including *Atp1b2*, *Aqp4, Aldh1l1, Aldoc, Apoe, Slc1a2* and *Slc1a3*, and *S100β* showed overall lower expression in culture compared to directly isolated samples, with no stage specific differences. *Gfap*, however, was markedly higher across all cultured conditions, suggesting that culture conditions foster a more reactive astrocyte phenotype. In line with this, stem cell and glial progenitor associated genes, such as *Vim* and *Nestin*, and cell cycle markers, such as *Ki-67, Mcm2, Cdk1, Top2a* and *Ccnb1* were robustly elevated across all cultured astrocytes, highlighting the strong proliferative influence of cultured conditions. Together, these expression profiles reveal that cultured postnatal astrocytes maintain core identity markers, but transition into a culture-induced proliferative and potential reactive gliosis state that diverges from the developmental trajectory of the directly isolated astrocytes. Still, DEG patterns revealed clear stage-specific developmental distinctions (Figure 3D). p3cultured astrocytes expressed neurodevelopmental genes including *Ccna1, Crmp1, Stmn3, Dcc* and *Cacna1c*, reflecting an immature state that retains transcriptional features of early postnatal development. p10cultured astrocytes upregulated transitional synaptogenic and cytoskeletal associated genes such as *Tns1, Sorbs1, Tmod2, Prkca* and *Rhou*, reflecting a transitional state characterized by emerging synaptic associated and cytoskeletal remodeling features. p14cultured astrocytes displayed a transcriptional profile enriched for *Idh3a, Got1, Nptx2, Cavin2* and *Angpt2*, reflecting a more mature state associated with enhanced metabolic and signaling specialization. Since the heatmap captures only the most distinctive DEGs, we extended our analysis to GO enrichment of all differentially expressed genes to define the major functional programs characterizing each stage (Figure 3E). p3cultured astrocytes were dominated by biosynthetic and RNA-processing pathways, including ribonucleoprotein complex biogenesis, ribosome biogenesis and RNA splicing. p10cultured astrocytes showed enrichment for actin filament organization, regulation of synapse structure or activity and regulation of synapse organization and extracellular matrix organization, consistent with a transitional stage marked by synaptic, cytoskeletal and extracellular matrix remodeling. p14cultured astrocytes were enriched for ribonucleoprotein complex biogenesis, ribosome biogenesis and rRNA metabolic processes, indicating elevated translational capacity accompanying progression toward a more mature astrocyte state. Overall, these data outline a developmental progression in which astrocytes transition from a highly proliferative early state at p3cultured, through a p10cultured stage marked by emerging synaptic-associated and cytoskeletal programs to a later p14cultured state showing enrichment of RNA- and ribosome-related pathways.

### Astrocytes from early, mid and late postnatal time points express a stage-specific protein secretome

Eventually, we aimed to identify factors driving gliogenic versus neurogenic potential of NSPCs. Mining our transcriptomic data set for potentially secreted factors, we identified that p3cultured astrocytes predominantly expressed calcium-binding proteins, extracellular matrix components (ECM), extracellular region proteins and hormone-like factors, reflecting a restricted early secretory repertoire (Figure 3F). In contrast, p10cultured astrocytes expressed the broadest and most diverse set of potential secreted molecules among the three stages, including multiple ECM-associated categories, lipid- and transport-related factors, enzymes, neuropeptide-related proteins, immune-associated factors, proteases and protease inhibitors and additional extracellular region proteins. p14cultured astrocytes displayed a more specialized profile of potential secreted factors enriched for transport- and metabolism-related categories, growth-factor-binding proteins, and several ECM-associated categories, cytokines, Notch ligand signaling components and angiogenesis-associated factors. These stage-specific shifts in potential secreted molecules mirror the transcriptional programs described above and reflect the gradual functional maturation of astrocytes under culture conditions. Importantly, it represents a valuable source of candidate factors retaining the potential to impel glial or neuronal differentiation of NSPCs.

### The *in vitro* environment induces a transcriptional state that diverges from *in vivo* astrocyte maturation

An intriguing, yet unresolved question was whether astrocyte continue their *in vivo* maturation program or instead adopt a culture-induced transcriptional state. Therefore, we compared the directly isolated p3, p10 p14 astrocytes with their corresponding *in vitro* populations. Principal component analysis revealed a clear separation between the direct and the cultured astrocytes at all stages. While the directly isolated astrocytes separated well according to the time point of isolation, the cultured astrocytes appeared rather intermingled (Figure 4A). A global heatmap further highlighted the formation of two major transcriptional clusters, the direct and the cultured, while also showing preserved stage-specific grouping within each condition, confirming that developmental signatures remain detectable even under culture conditions (Supplementary Figure 2).

**Figure 4:**
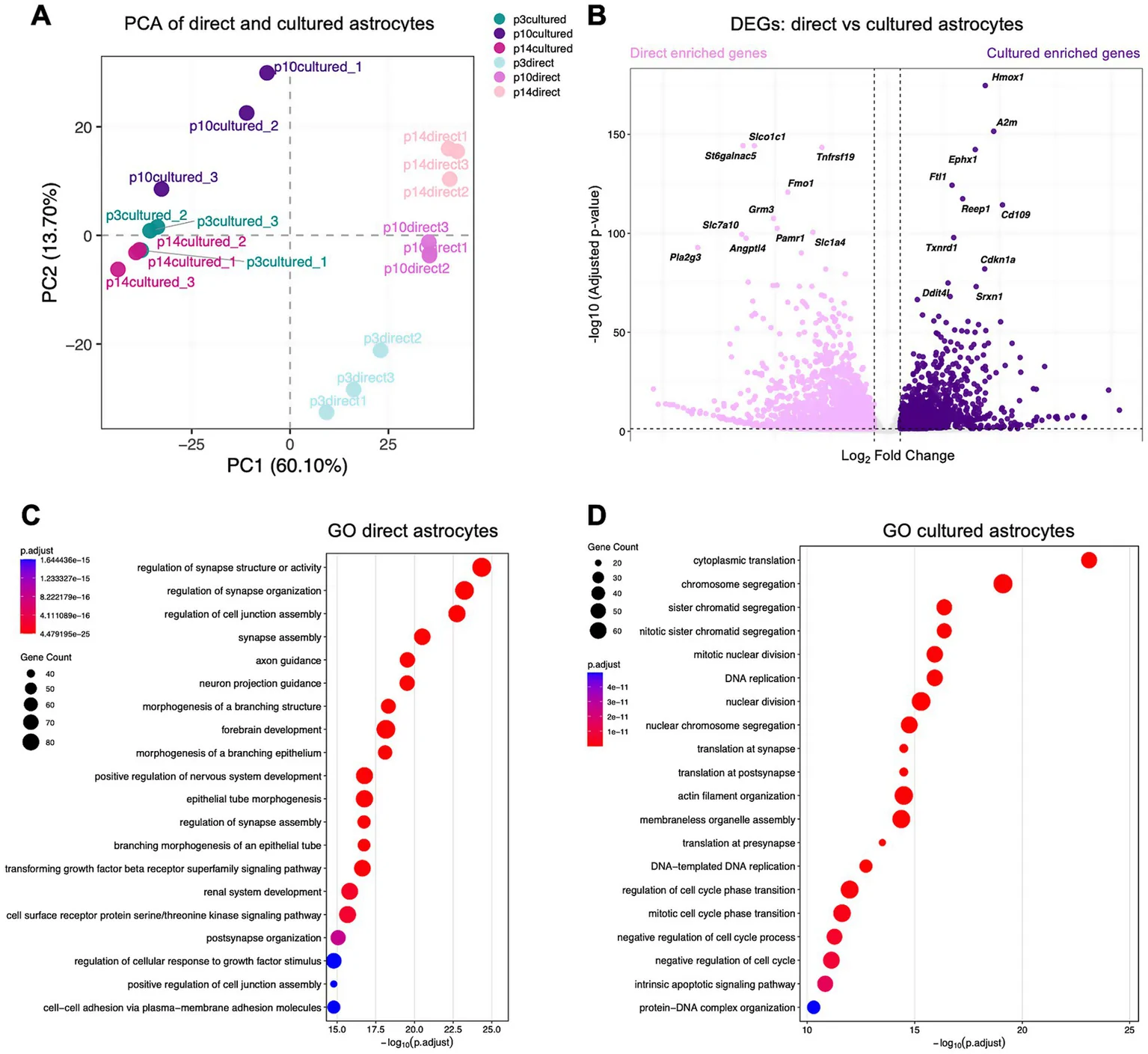
Transcriptional differences between directly isolated and cultured hippocampal astrocytes. **(A)** Principal Component Analysis (PCA) of directly isolated and cultured astrocytes at p3, p10 and p14. **(B)** Volcano plot of DEGs from direct astrocytes and cultured astrocytes. **(C)** GO enrichment analysis of genes enriched in directly isolated astrocytes. **(D)** GO enrichment analysis of genes enriched in cultured astrocytes.

Differential expression analysis revealed a set of DEGs conserved across all developmental timepoints driving the robust transcriptional separation between direct and cultured astrocytes (Figure 4B). Specifically, directly isolated astrocytes upregulated physiological and metabolic transporter genes such as *Slc7a10, Slco1c1* and *Grm3*. In contrast, cultured astrocytes were characterized by a prominent upregulation of core stress-adaptation, antioxidant and cell-cycle checkpoint genes, including *Hmox1, Cdkn1a, Srxn1* and *Txnrd1.* Placing these molecular changes into a broader functional perspective, GO enrichment analysis demonstrated that directly isolated astrocytes showed strong enrichment for regulation of synapse structure or activity, synapse assembly and axon guidance (Figure 4C). In contrast, the broader transcriptional signature of cultured astrocytes was dominated by pathways related to chromosome segregation, DNA replication and nuclear division (Figure 4D). Together, these analyses show that *in vitro* maturation diverges from the *in vivo* developmental trajectory and results in a distinct culture-specific transcriptional state.

### Metabolomic profiling reveals stage-specific metabolic signatures in astrocyte conditioned media and identifies metabolites that modulate NSC differentiation *in vitro*

The transcriptional analyses revealed that the progression along postnatal stages was accompanied by increased metabolic, enzymatic and secretory pathway activity, indicating that p3 and p14 astrocytes might generate distinct metabolic environments. Since transcriptomics provide only indirect proof, we directly compared the secreted metabolites by performing untargeted metabolomic profiling of ACM collected from p3 and p14 astrocytes across four mass-spectrometry modes (Figure 5A). We specifically focused on only two conditions as ACM from p3 revealed a strong gliogenic effect onto NSPCs, whereas ACM from p14 astrocytes exhibited the clearest neurogenic potential.

**Figure 5:**
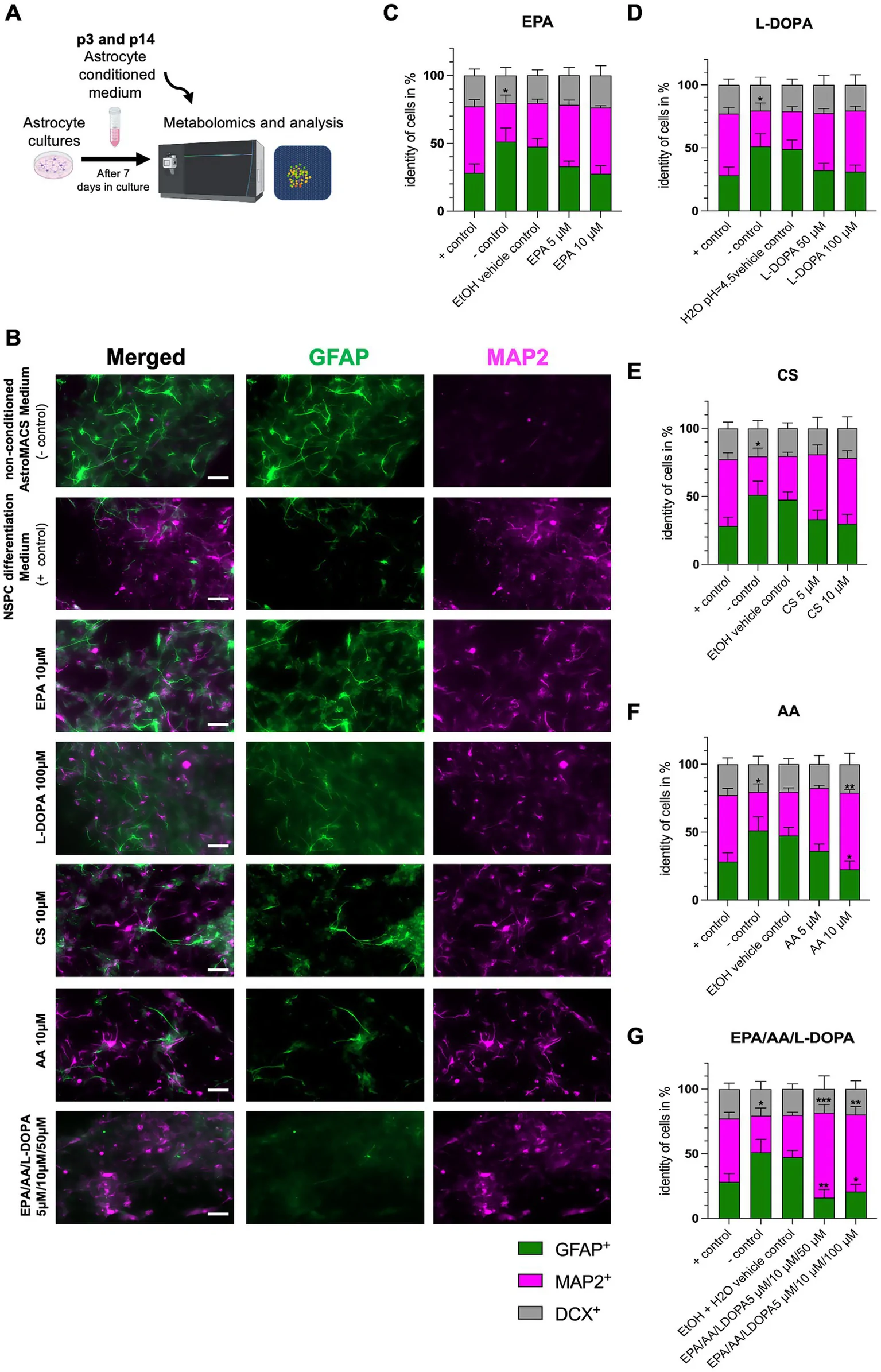
Metabolomic profiling of astrocyte conditioned media and metabolite tested on adult NSPCs *in vitro*. **(A)** Schematic of the experimental design. p3 and p14 astrocytes were cultured for 7 days, and ACM was collected for metabolomics. **(B)** Immunostainings of adult NSPCs treated with selected metabolites, labeled for MAP2, GFAP, DCX and DAPI. **(C–F)** Dose–response curves for EPA, L-DOPA, CS and AA, respectively. **(G)** Dose–response curves for EPA/AA/L-DOPA combinations. Scale bars = 50 μm. Masouti, E. (2026) https://BioRender.com/9rx83j7

Of the 354 detected metabolites, 101 showed at least a two-fold enrichment relative to control medium (non-conditioned AstroMACS medium), ensuring that only metabolites actively secreted by the astrocytes were retained for analysis. From those 101 metabolites, 91 were enriched exclusively in p3 ACM and 10 were enriched exclusively in p14 ACM. With the aim to identify potentially neurogenic metabolites, we focused on these 10 factors, and selected arachidonic acid (AA), eicosapentaenoic acid (EPA), L-DOPA and cholesterol sulfate (CS) for functional testing in adult NSPC cultures using two different concentrations and several combinations (Supplementary Figure 3). Immunostainings for MAP2, GFAP and DCX revealed metabolite-specific effects on neuronal and glial differentiation across the tested conditions (Figure 5B). Across all effective combinations, DCX-positive cells remained unchanged. A heatmap summarizing all tested metabolites and concentrations revealed distinct glial and neuronal differentiation signatures (Supplementary Figure 3B). As negative controls, we used NSPCs grown in non-conditioned AstroMACS Medium (28.3% MAP2^+^ neurons, 51.2% GFAP^+^ astrocytes, and 20.5% DCX^+^ immature neurons; Figure 5B; Supplementary Figures 3A,B) as well as vehicle controls for EtOH and acidified water, which served as dissolvents of the metabolites (Supplementary Figure 3). NSPCs grown in NSPC differentiation medium served as positive controls (49.0% MAP2^+^ neurons, 28.2% GFAP^+^ astrocytes, and 22.8% DCX^+^ immature neurons; Figure 5B; Supplementary Figure 3B). The addition of EPA, L-DOPA and CS (in their respective highest concentration) to the AstroMACS Medium induced neuronal differentiation at levels comparable to NSPC differentiation medium (Figure 5C–E), indicating that all three metabolites alone are potent enough to drive neuronal differentiation from NSPCs. The addition of 10 μM AA even exceeded the NSPC’s neurogenic capacity, and resulted in 56.4% of MAP2^+^ neurons, while reducing GFAP^+^ cells to 22.5% (Figure 5F). The combination of EPA (5 μM)/AA (10 μM)/L-DOPA (50 μM) was the strongest inducer of neuronal differentiation (65.7% MAP2^+^ neurons, 16.1% GFAP^+^ astrocytes; Figure 5G; Supplementary Figure 3B), outperforming the premixed NSPC differentiation medium by more than 15%.

Together, we have shown that p14 astrocyte release metabolites, which are specific and potent inducers of neuronal differentiation from NSPCs, implicating astrocytic metabolites as powerful regulators of neurogenic potential.

## Discussion

Astrocytes orchestrate hippocampal development by providing trophic factors, metabolic substrates and extracellular-matrix cues that shape the neurogenic niche. By isolating hippocampal astrocytes at p3, p10 and p14, we captured key developmental stages of astrocyte maturation that accompany granule neuron differentiation. Integrated transcriptomic and metabolomic profiling revealed that although astrocytes in culture acquire a shared molecular profile imprinted by culture conditions, they retain biologically relevant, stage-specific molecular signatures. Interestingly, astrocyte at different developmental stages release a specific cocktail of factors to promote either neurogenesis or gliogenesis. Conditioned medium collected from p10 and p14 astrocytes markedly increased neuronal differentiation of adult hippocampal NSPCs, whereas p3 promoted a gliogenic program, indicating a shift in the astrocyte secretome along postnatal development. Metabolomics from p3 and p14 ACM revealed novel astrocyte-released metabolites, including arachidonic acid, cholesterol sulfate, eicosapentaenoic acid, and L-DOPA, which significantly enhanced the neurogenic potential of NSPCs in culture. Together, these findings establish developing astrocytes as key drivers of the hippocampal development and reveal previously unrecognized astrocyte-derived metabolic cues that actively shape the neurogenic niche.

Supporting the view that astrocyte maturation involves coordinated transcriptional, metabolic and functional changes, bulk-RNA seq of directly isolated hippocampal astrocytes showed a clear developmental progression shifting from an immature, proliferative state at p3 to lipid-metabolic and synaptogenic programs at p10, and leading into a mature mitochondrial and neuro-supportive profile at p14 (Molofsky and Deneen, 2015). Conserved metabolic shifts have been described in the cortex and spinal cord, where astrocytes rely on mitochondrial oxidative phosphorylation and fatty acid oxidation to first sustain proliferation, and subsequently acquire enhanced synaptogenic and homeostatic functions as they mature (Eraso-Pichot et al., 2018; Morant-Ferrando et al., 2023; Bolaños and Almeida, 2026). The pronounced metabolic reorganization at p10, marked by enrichment of lipid metabolism pathways, likely reflects a transient phase supporting membrane synthesis and active synaptogenesis (Mauch et al., 2001; Pfrieger, 2003; Allen et al., 2012). By p14, the dominance of oxidative phosphorylation establishes a metabolically specialized state tailored to sustain neuronal activity and homeostasis (Eraso-Pichot et al., 2018; Hasel et al., 2017; Morant-Ferrando et al., 2023; Bolaños and Almeida, 2026). Together, these observations indicate that directly isolated astrocytes accurately captured *in vivo* maturation states, and provide a framework for understanding how astrocyte maturation and neuronal maturation are adjusted to form and refine hippocampal circuits.

Across all developmental stages, cultured astrocytes adopted a shared culture-induced transcriptional program that diverged from *in vivo* maturation, while retaining signatures of their original developmental stage. This divergence reinforced the idea that astrocyte maturation is not cell-autonomous, but depends on their direct cellular, structural and molecular environment (Molofsky and Deneen, 2015; Allen and Lyons, 2018; Khakh and Deneen, 2019; Hasel et al., 2017). Nevertheless, *in vitro* astrocytes continued to express stage-defining genes characteristic of their *in vivo* counterparts, suggesting that intrinsic transcriptional and epigenetic memory persists for at least a week in culture (Molofsky and Deneen, 2015; Hasel et al., 2017). Without the appropriate environmental cues though, this memory was insufficient to maintain normal maturation, leading to the upregulation of proliferative and stress-adaptive pathways (Foo et al., 2011; Hasel et al., 2017). Together, these findings indicate that removal from the hippocampal niche interrupts the normal maturation trajectory, while astrocytes still preserve a transcriptional imprint of their original *in vivo* state.

The stage-specific potential secretome profiles provide insight into how developing hippocampal astrocytes shape their extracellular environment as they progress toward functional maturation. Early p3cultured astrocytes predominantly express transcripts encoding ECM components and calcium-binding proteins, supporting perinatal extracellular matrix organization and initial preparation of the synaptic milieu during the first postnatal days (Wiese et al., 2012). By p10, the potential secretome broadens to include proteases, lipid-associated molecules and neuropeptide precursors, reflecting a transitional state in which astrocytes activate signaling pathways associated with synapse formation, refinement and circuit assembly, processes known to peak between p7 and p21 (Wiese et al., 2012; Molofsky and Deneen, 2015). In contrast, p14cultured astrocytes display a more specialized repertoire enriched for growth-factor-binding proteins, cytokine-related molecules, Notch-associated ligands and angiogenesis-linked factors, reflecting mature homeostatic functions driven neuron-derived extrinsic cues such as cytokines, BMPs, and Notch ligands during late postnatal development (Cahoy et al., 2008; Zhang et al., 2014; Markey et al., 2023). Importantly, many of the identified potential secreted molecules, including ECM components, synaptogenic cues and metabolic regulators correspond to well-established astrocyte-derived signals known to shape neuronal development. Classic factors such as Hevin, thrombospondins and glypicans promote structural and functional synapse formation during the postnatal period (Christopherson et al., 2005; Kucukdereli et al., 2011; Allen et al., 2012), highlighting that several of the molecules detected in our dataset are directly linked to circuit assembly *in vivo* (Singh et al., 2016).

With the aim to identify novel astrocyte-secreted factors promoting neurogenesis, we performed metabolomic profiling of the ACM from p3 and p14 astrocytes inducing either glial (p3) or neuronal differentiation (p14) in hippocampal NSPC cultures. Here, we observed that p3 astrocytes released a broad and abundant spectrum of metabolites consistent with their highly proliferative, biosynthetically active state and the metabolic plasticity characteristic of immature glia (Cahoy et al., 2008; Zhang et al., 2014; Zehnder et al., 2021; Zhang et al., 2022). p3 astrocytes secrete a diverse repertoire of amino-acid, nucleotide and carbon metabolites expanding the classical view of astrocytic support of neurons via the lactate shuttle (Pellerin and Magistretti, 1994). This metabolic flexibility aligns with transcriptomic evidence that early postnatal astrocytes retain stem-cell-like features and engage glycolysis, the pentose-phosphate pathway and the TCA cycle to generate energy and building blocks for growth and biosynthesis (Cahoy et al., 2008; Zhang et al., 2014; Zehnder et al., 2021; Zhang et al., 2022). As astrocytes mature, their secreted metabolite cocktail becomes more selective, exhibiting a pronounced enrichment for lipid-derived and catecholamine-related molecules, including polyunsaturated fatty acids (PUFAs) such as AA and EPA, L-DOPA and cholesterol sulfate. This pattern mirrors *in vivo* reports that maturing astrocytes undergo a metabolic transition characterized by increased mitochondrial activity and enhanced fatty acid β-oxidation during synaptogenesis (Eraso-Pichot et al., 2018; Morant-Ferrando et al., 2023; Bolaños and Almeida, 2026). The enrichment of PUFAs in p14 ACM is consistent with evidence showing that both omega-3 and omega-6 PUFAs modulate NSPC behavior and neuronal differentiation. In particular, omega-3 s such as Docosahexaenoic acid (DHA) and EPA promote neuronal differentiation by downregulating Hes1 and upregulating activator-type bHLH transcription factors including Hes6, NeuroD and Mash1, thereby enhancing MAP2 and cell cycle inhibitors like p21 and p27 (Katakura et al., 2009). Beyond these omega-3 mechanisms, both DHA and the omega-6 PUFA AA increase the number of neurogenic NSCs during postnatal development (Sakayori et al., 2011). While classical work suggests that AA primarily supports NSC maintenance, our data indicate that AA promotes neuronal differentiation and maturation, pointing to a context-dependent role of AA in hippocampal development. In addition, cholesterol sulfate, also enriched at p14, exerts neuroprotective and anti-apoptotic effects, potentially via metabolic support, while catecholamine related metabolites like L-DOPA modulate oxidative stress responses and dopaminergic lineage specification (Meiser et al., 2013; Prah et al., 2019). Together, these observations support a model in which early astrocytes provide broad biosynthetic support, whereas more mature astrocytes provide specialized secreted cues capable of modulating hippocampal differentiation. Our metabolomic profiling thus reveals a previously unrecognized shift toward lipid- and catecholamine-enriched astrocyte-secreted metabolic environment, highlighting the combinatorial impact of PUFAs, cholesterol sulfate and L-DOPA in coordinating neural lineage decisions and neuronal maturation.

In addition to metabolic cues, classical morphogen pathways may contribute to the proneurogenic effects of the maturing astrocyte secretome. Astrocyte-derived Wnt/beta-catenin signaling is a well-established regulator of adult hippocampal neurogenesis (Lie et al., 2005), acting primarily on the early steps of neurogenesis, including neuroblast proliferation and neuronal fate commitment. In our transcriptomic dataset, *Wnt3* and *Wnt3a* were expressed at or below the detection threshold in both directly isolated and cultured astrocytes, suggesting that these ligands are unlikely to mediate the pro-maturational effects observed here. Notably, however, *Wnt7a* and *Wnt7b* were abundantly expressed in our astrocyte preparations across all developmental stages. Interestingly, expression of *Wnt7a* peaks in p10 astrocytes the synaptogenic window during which astrocyte maturation and circuit formation proceed in parallel. Previous work identified an important role for *Wnt7a* in neuronal differentiation and maturation via beta-catenin-neurogenin 2 signaling and regulation of dendritic development of newborn granule neurons in the dentate gyrus (Qu et al., 2013). Furthermore, Wnt7-family signaling regulates axonal remodeling and synapse formation (Hall et al., 2000; Ahmad-Annuar et al., 2006). Given its function, *Wnt7a* as an astrocytic secreted factor is a likely contributor to neuronal maturation.

The present study reveals the following limitation: First, the astrocytes used for bulk RNA-seq and contain a mixture of astrocytes in different developmental stages. While at p3 we harvest mostly NSPCs, p10 and p14 contain both more mature superficial astrocyte and the less mature deep layer astrocytes. Second, because 6–8 animals were pooled per age group to obtain sufficient cell numbers, our analyses capture population-level averages and cannot resolve heterogeneity between individual animals or between astrocyte subpopulations. Third, the ACM used for our functional assays was collected from astrocytes maintained in culture for 7 days. Although we show that cultured astrocytes retain stage-specific transcriptional signatures, culture induces proliferative and stress-adaptive programs that differ from the *in vivo* environment. Consequently, our data demonstrate that stage-dependent signals that promote neuronal maturation are present in the hippocampal astrocyte secretome, although our approach cannot determine which specific astrocyte subpopulations produce them. Future single-cell and spatial transcriptomic approaches would be required to resolve which astrocyte subsets produce the pro-neurogenic cues identified here.

Overall, our findings position astrocytes as active instructors of postnatal brain development. Rather than serving solely as supportive elements, astrocytes shape both their own maturation program as well as their direct environment through a combination of transcriptional programs, metabolic activity and secreted cues. By supplying factors that influence NSC behavior and neuronal differentiation, astrocytes help in guiding the trajectory of hippocampal circuit formation. These insights reveal a developmental framework in which astrocytes progressively refine their regulatory repertoire, suggesting that shifts in glial states and metabolism represent key mechanisms guiding neuronal maturation and refinement.

## Data Availability

The datasets presented in this study can be found in online repositories. The names of the repository/repositories and accession number(s) can be found in the article/Supplementary material.
