# Supplementary material for: Astrocyte maturation creates a stage-specific niche that instructs hippocampal neurogenesis
Source: Front Cell Neurosci. 2026 Oct 7;20:1966983. doi: 10.3389/fncel.2026.1966983 (PMC13682960; doi:10.3389/fncel.2026.1966983)
Supplement: Supplementary file 1 [file Data_Sheet_1.ZIP › Supplementary Data/Supplementary Figures/Supplementary Figure Legends.docx]

**Supplementary Figure 1: Serum exposure induces morphological and cytoskeletal changes in hippocampal astrocytes.** (A) Brightfield and GFAP immunofluorescence images of astrocytes cultured in serum-containing medium. Serum exposure results in a flattened, elongated morphology and thick GFAP-positive processes. (B) Brightfield and GFAP immunofluorescence images of astrocytes cultured in serum-free medium. Astrocytes retain a star-like morphology and exhibit fine GFAP-positive processes.

**Supplementary Figure 2. Global transcriptional landscape of directly isolated and cultured astrocytes.** Heatmap showing hierarchical clustering of all expressed genes across directly isolated (p3, p10, p14) and cultured astrocytes derived from the same developmental stages.

**Supplementary Figure 3. Extended metabolite screen and pathway analysis for p3 ACM.** (A) Full immunostaining panels (GFAP, MAP2, DCX) for all metabolites and concentrations tested. (B) Heatmap listing MAP2-, GFAP- and DCX-positive cell percentages across all metabolites and concentrations tested.
