# Supplementary material for: Astrocyte maturation creates a stage-specific niche that instructs hippocampal neurogenesis
Source: Front Cell Neurosci. 2026 Oct 7;20:1966983. doi: 10.3389/fncel.2026.1966983 (PMC13682960; doi:10.3389/fncel.2026.1966983)
Supplement: Supplementary file 1 [file Data_Sheet_1.ZIP › Supplementary Data/Supplementary Tables 1 and 2/Supplementary Table Legends.docx]

**Supplementary Table 1: GO enrichment analysis of shared and stage-enriched genes in directly isolated hippocampal astrocytes.**

**Supplementary Table 2: GO enrichment analysis of shared and stage-enriched genes in cultured hippocampal astrocytes.**
